# Do Hospitalized Premature Infants Benefit from Music Interventions? A Systematic Review of Randomized Controlled Trials

**DOI:** 10.1371/journal.pone.0161848

**Published:** 2016-09-08

**Authors:** Marianne J. E. van der Heijden, Sadaf Oliai Araghi, Johannes Jeekel, Irwin K. M Reiss, M. G. Myriam Hunink, Monique van Dijk

**Affiliations:** 1 Department of Paediatrics, Erasmus MC, Rotterdam, the Netherlands; 2 Department of Neuroscience, Erasmus MC, Rotterdam, the Netherlands; 3 Department of Neonatology, Erasmus MC, Rotterdam, The Netherlands; 4 Department of Epidemiology, Erasmus MC, Rotterdam, the Netherlands; 5 Department of Radiology, Erasmus MC, Rotterdam, the Netherlands; 6 Department of Health Policy and Management, Harvard T.H. Chan School of Public Health, Boston, MA, United States of America; 7 Department of Paediatrics, division of Neonatology and Department of Pediatric Surgery, Intensive care Erasmus MC, Rotterdam, the Netherlands; University of Exeter, UNITED KINGDOM

## Abstract

**Objective:**

Neonatal intensive care units (NICU) around the world increasingly use music interventions. The most recent systematic review of randomized controlled trials (RCT) dates from 2009. Since then, 15 new RCTs have been published. We provide an updated systematic review on the possible benefits of music interventions on premature infants’ well-being.

**Methods:**

We searched 13 electronic databases and 12 journals from their first available date until August 2016. Included were all RCTs published in English with at least 10 participants per group, including infants born prematurely and admitted to the NICU. Interventions were either recorded music interventions or live music therapy interventions. All control conditions were accepted as long as the effects of the music intervention could be analysed separately. A meta-analysis was not possible due to incompleteness and heterogeneity of the data.

**Results:**

After removal of duplicates the searches retrieved 4893 citations, 20 of which fulfilled the inclusion/exclusion criteria. The 20 included studies encompassed 1128 participants receiving recorded or live music interventions in the NICU between 24 and 40 weeks gestational age. Twenty-six different outcomes were reported which we classified into three categories: physiological parameters; growth and feeding; behavioural state, relaxation outcomes and pain. Live music interventions were shown to improve sleep in three out of the four studies and heart rate in two out of the four studies. Recorded music improved heart rate in two out of six studies. Better feeding and sucking outcomes were reported in one study using live music and in two studies using recorded music.

**Conclusions:**

Although music interventions show promising results in some studies, the variation in quality of the studies, age groups, outcome measures and timing of the interventions across the studies makes it difficult to draw strong conclusions on the effects of music in premature infants.

## Introduction

Health professionals are increasingly aware that the acoustic environment in the neonatal intensive care unit (NICU) may affect infants’ well-being. Where unpredictable noise adversely affects sleep and physiologic stability [1,2], meaningful auditory stimulation, such as music, might contribute to the neurodevelopment of premature infants.

Music is defined as intentional sound described in terms of pleasing harmonies, dynamics, rhythm, tempo and volume [3]. Music interventions can consist of a combination of instrumental music and song, performed live or pre-recorded. Music interventions for the NICU should be soothing and not use too many different elements in terms of instruments, rhythms, timbres, melodies and harmonies [4]. The preferred choice of music is a lullaby, softly sung or played on an instrument. Several observational studies suggest that music might have a positive effect on physiological parameters, feeding and development of premature infants [5–13].

*Recorded* music interventions in the NICU usually consist of music softly played through an audio player in or outside the incubator. This is recommended for infants from 28 weeks gestational age (GA) [14]. Another recorded music intervention is the pacifier activated lullaby (PAL), recommended for infants from 30 weeks GA [14], where the infants’ sucking on a pacifier activates a lullaby played inside the incubator. In *live* music therapy interventions a certified music therapist softly sings lullabies, sometimes accompanied by guitar, harp, or drum playing. Other instruments used are the Gato Box, a 2- or 4-tone wooden box or drum that is played with the fingers, and the Ocean Disc, an instrument shaped as a round disc with metal beads inside that make a whooshing sound to the padded interior shell of the disc. Live music therapy in the NICU is recommended for infants from 32 weeks GA [14].

Up to August 2016, nine reviews on the effects of music interventions in premature infants have been published, [3,14–21] not all of which included only RCTs. Standley et al. [14,21] published an updated meta-analysis in 2012 concluding that music interventions have a beneficial significant effect on heart rate, behavioural state, oxygen saturation, sucking/feeding ability and length of stay. Apart from RCTs, the meta-analysis also included non-randomized trials and studies with samples sizes <10 per group. Hartling et al. [16] published the most recent systematic review of only RCTs on the effects of music in neonates in 2009, including both preterm and term infants. The authors concluded that music may be beneficial on behavioural state, physiological measures and pain, but the heterogeneity in studies precluded a meta-analysis and definite conclusions on efficacy. Since 2009, fifteen new RCTs on this topic have been published, which justifies our update. This current systematic review of RCTs on the effectiveness of live and recorded music interventions in premature infants in the NICU was performed with no restrictions on type of outcome measures.

## Methods

This systematic review followed the recommendations of the Cochrane Collaboration and the PRISMA Guidelines for reporting a systematic review (see S1 Table for the PRISMA checklist). We made use of a pre-defined research protocol (see S1 File Review protocol).

### Criteria for Considering Studies for This Review

The following inclusion criteria were applied: papers published in English reporting RCTs including prematurely born infants 24–37 weeks GA with a parallel group, crossover or cluster design. Only studies in which the effects of music could be analysed separately from the control condition were included. Studies in which fewer than 10 patients received the intervention were considered pilot studies and were therefore excluded. Interventions were either *recorded* or *live* music interventions as defined above. Interventions that required participation of a parent were only accepted if it included musical expression such as singing. Excluded were studies using speech or the sounds of the womb, and studies with interventions that used non-human sounds, such as nature sounds.

### Search Methods for Identification of Studies

We searched 13 electronic databases and trial registers from their first available date until August 2016. Furthermore we hand-searched 12 journals from their first available date (see S2 File Full list of search terms, electronic databases and hand-searched journals). The reference lists of the articles were checked for other relevant articles not retrieved by the search strategies, and attempted to order full-text articles when necessary. Additionally, we defined key references (see S2 File Full list of search terms, electronic databases and hand-searched journals) and performed a forward citation search in Web of Science.

### Data Collection

Two authors (MvdH and SO) selected the potentially eligible articles by independently screening the titles and abstracts of the retrieved records for relevance. A study was rejected if the abstract made clear that the trial did not meet the inclusion criteria. If there was doubt about the inclusion the two independent reviewers would consult the final author (MvD). Two authors extracted data for all articles using the Cochrane Collaboration Data Collection Form for intervention reviews (see S3 File Cochrane data collection form for intervention review: RCTs). The results of the data extraction were compared between the two authors to preclude any differences. Risk of bias was assessed using the Cochrane Risk of Bias tool. Items scored included: random sequence generation, allocation concealment, blinding of participants and personnel, blinding of outcome assessment, incomplete outcome data and selective reporting.

### Data Presentation and Analysis

**Fig 1:**
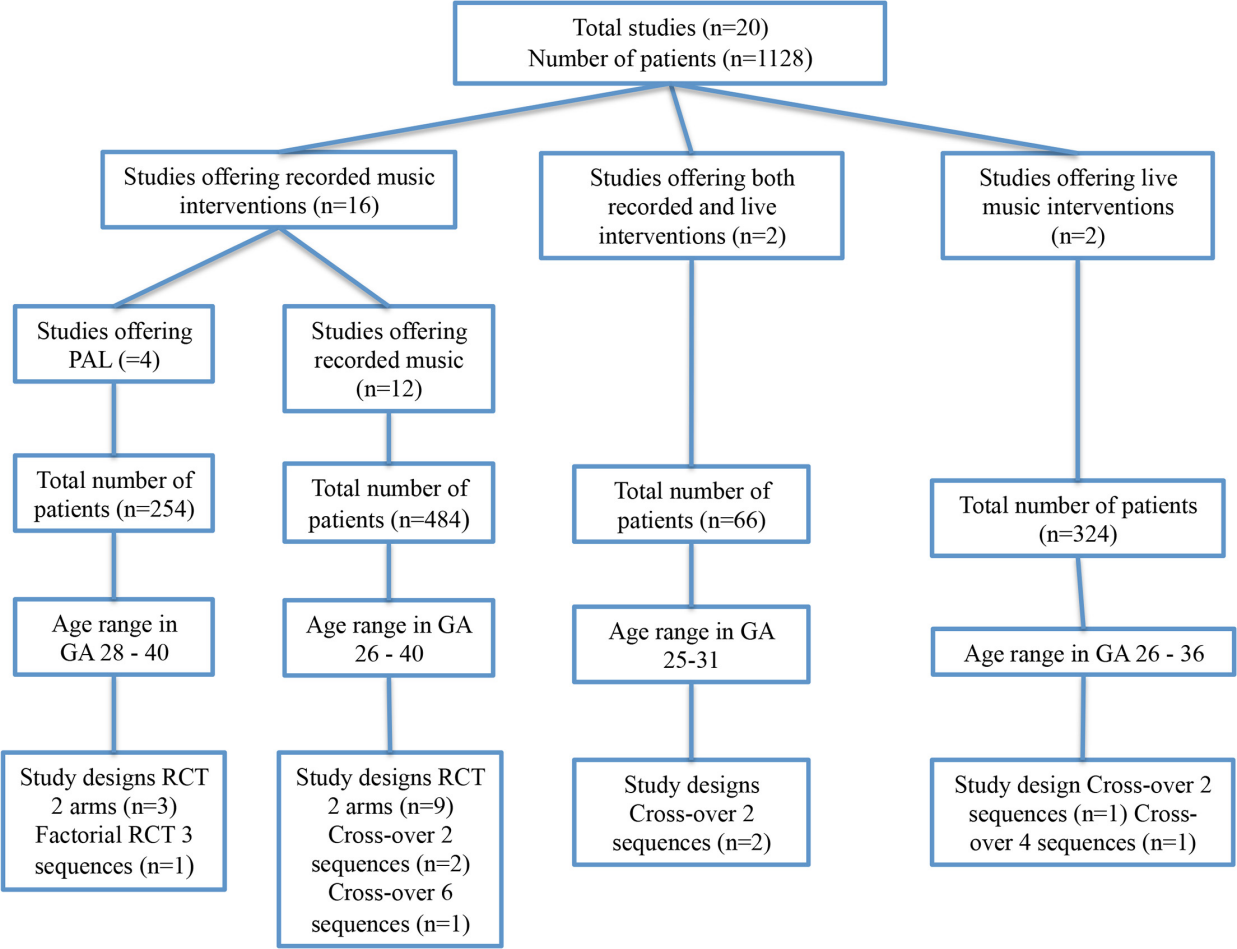
Characteristics of included studies.

**Fig 2:**
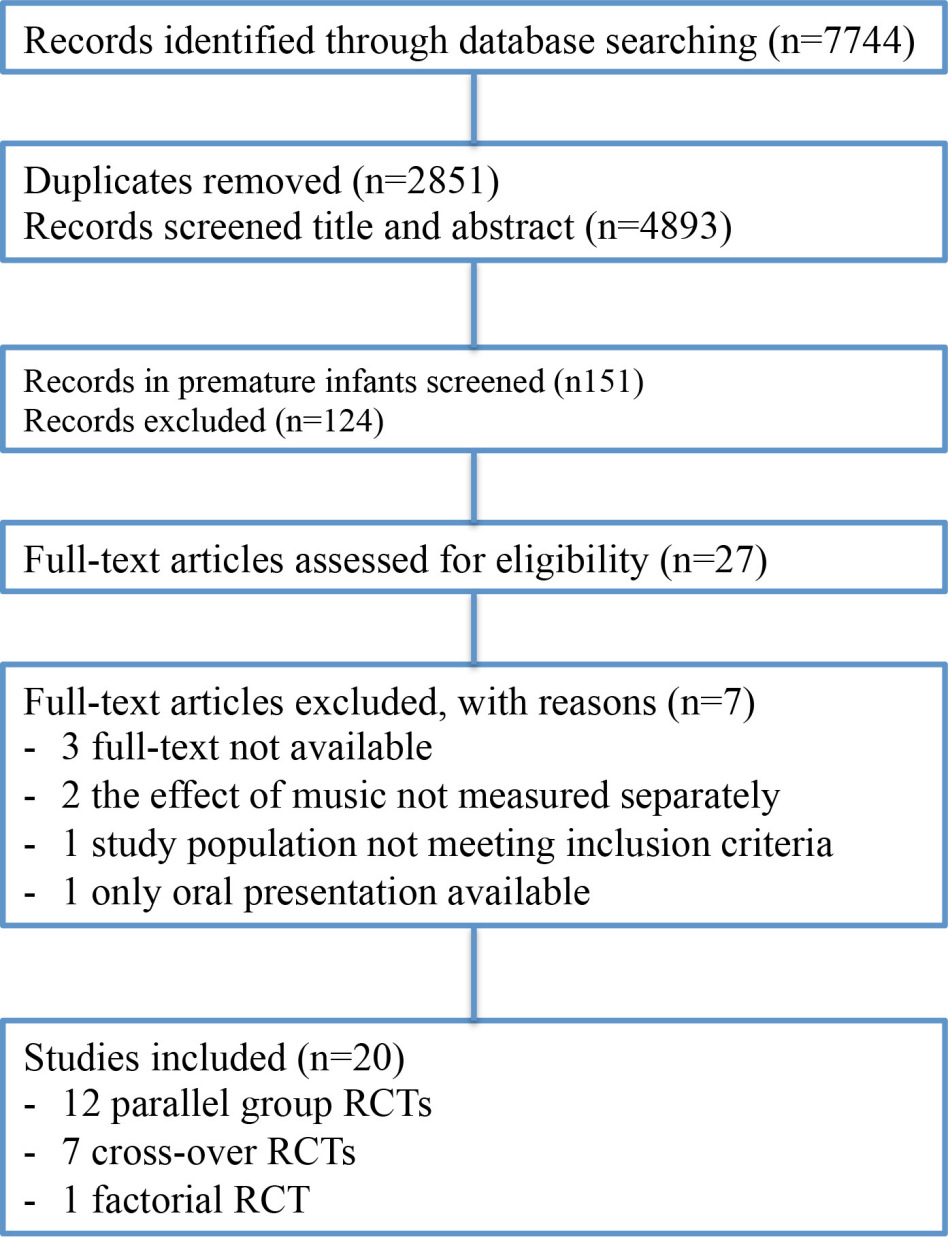
PRISMA flowchart.

We classified the 26 different outcomes measured in the studies into three categories: physiological parameters; growth and feeding; behavioural state, relaxation outcomes and pain. A meta-analysis was not possible due to missing and heterogeneous data. For the purpose of this review we only present the results compared between the intervention and control groups. Fig 1 gives the characteristics of the included studies (see S2 Table for the extensive overview); Fig 2 depicts the number of records identified, included and excluded, and the reasons for exclusion in the PRISMA Flowchart. Table 1 shows the risk of bias for all studies (determined with the Cochrane Collaboration tool for assessing risk of bias in included studies [22]; and Table 2 the characteristics of the music interventions. Tables 3, 4 and 5 show the between group results for the respective outcome categories mentioned above.

**Table 1:** Risk of bias. Cochrane Collaboration tool for assessing risk of bias.

Author	Selection bias		Performance bias	Detection bias	Attribution bias	Reporting bias	Overall risk of bias
	Random sequence generation	Allocation concealment	Blinding participants and personnel	Blinding outcomes	Incomplete outcome data	Selective	
Wirth (2016)	Unclear	Unclear	High	High	Unclear	Unclear	Unclear
Jabraeili (2016)	Low	Low	Low	Unclear	Unclear	Unclear	Unclear
Cardoso (2014)	Low	Low	Unclear	Unclear	Unclear	Unclear	Unclear
Chorna (2014)	Low	Unclear	Low	Low	Low	Low	Low
Dorn (2014)	Low	Low	Unclear	Unclear	Low	Low	Unclear
Garunkstiene (2014)	Low	Unclear	Unclear	Low	Low	Low	Unclear
Amini (2013)	Low	Unclear	High	Unclear	Unclear	Unclear	High
Loewy (2013)	Low	Low	Low	Low	Low	Unclear	Low
Alipour (2012)	Low	Low	Unclear	Low	Low	Low	Low
Aydin (2012)	Unclear	Unclear	High	High	Low	Low	High
Olischar (2011)	Low	Low	Low	Low	Low	Low	Low
Shlez (2011)	Unclear	Unclear	Low	Low	Unclear	Low	Unclear
Farhat (2010)	Low	Unclear	Unclear	Unclear	Low	Unclear	Unclear
Lubetzky (2010)	Low	Unclear	Unclear	Unclear	Low	Low	Unclear
Standley (2010)	Low	Unclear	Unclear	Low	Low	Unclear	Unclear
Keith (2009)	Low	Unclear	Unclear	Unclear	Unclear	Unclear	High
Whipple (2008)	Unclear	Unclear	Unclear	Unclear	Unclear	Unclear	Unclear
Arnon (2006)	Low	High	Low	Low	Unclear	Unclear	Unclear
Calabro (2005)	Low	Low	Unclear	Low	Unclear	Low	Unclear
Standley(2003)	Unclear	Unclear	Unclear	Low	Low	Low	Unclear

Low risk of bias–it is plausible that any bias present is unlikely to seriously alter the results. Unclear risk of bias–too few details known to classify. High risk of bias–it is plausible there is bias that seriously weakens the confidence in the results

**Table 2:** Characteristics of music interventions per study. Characteristics of music interventions per study.

Author (year)	Music selection (selected by)	Type of delivery (location)	dB	Duration of study, length and frequency of intervention	Timing of intervention
**Recorded music**					
Wirth (2016)	Lullaby (researcher)	Speaker (in incubator)	55–65 dB	14 days, 30 mins, 1x daily	30–60 mins after feeding
Jabraeili (2016)	Brahm’s lullaby (researcher)	Speaker (NR)	65dB	3 days, 15 mins, 1x daily	Between 10AM and 7 PM
Cardoso (2014)	Lullaby (researcher)	Headphone with Mp4 player (in incubator)	NR	1 day, 10 minutes, 1x daily	10 minutes before arterial puncture
Chorna (2014)	Children’s songs sung by mother (researcher)	Pacifier Activated Lullaby (PAL) (in incubator)	NR	5 days, NR, 15 minutes	30–45 minutes before feeding
Dorn (2014)	Collection of lullabies (researcher)	Audio player and loudspeaker (in incubator)	55–65 dB	14 days, 1x daily, 30 minutes	Between 20.00 and 21.00 every evening
Garunkstiene (2014)*	Selection of Lithuanian and traditional Western lullabies (same for live and recorded) sung by female (music therapist)	Speakers at 30 cm from infant’s head (in incubator)	45–50 dB	3 days, 1x daily, 20 minutes	30 minutes after feeding
Amini (2013)	Live: Iranian Lullaby Recorded: Mozart Sonata K.448, Baby Mozart CD (researcher)	Speakers at 30 cm from infant’s head (in incubator)	45–50 dB	6 days, 1x daily for two days per intervention group, 20 minutes	1 hour after feeding
Alipour (2012)	Iranian Lullaby (researcher)	Headphone (in incubator)	50–60 dB	1 day, 1x daily, 20 minutes	30 minutes after the last feeding
Aydin (2012)	Classical music, not specified (researcher)	Two loudspeakers at the feet of the infant (in incubator)	40–65 dB	1 day till discharge (hospitalization 22–23 days), 1x daily, 1 hour	Afternoon
Olischar (2011)	Brahms Lullaby (researcher)	Speaker at 30 cm from infant’s head (in incubator)	50–55 dB	1 day, 1x daily, 20 minutes	After one Sleep-Wake-Cycle on aEEG
Farhat (2010)	Iranian Lullaby (music therapist)	MP3 player and headphones (in incubator)	60–65 dB	8 days, 1x daily, 20 minutes	30 minutes after feeding
Lubetzky (2010)	Mozart (researcher)	Mini CD device and speakers at 30 cm distance from infants’ ears (in incubator)	65–70 dB	2 days, 1x daily, 30 minutes	1 hour after the last feeding in the afternoon
Standley (2010)	Continuous selection of lullabies sung by female vocalist with minimal accompaniment (music therapist)	Pacifier Activated Lullaby (PAL) (in incubator)	65 dB	5 days, 1x or 3x daily, 15 or 45 minutes	From 4 to 5 o’clock in the afternoon
Keith (2009)	Lullaby: Female singing unaccompanied lullabies and songs for young children (music therapist)	CD player and speakers (in incubator)	<70 dB	4 days, 1x daily, 18 minutes	NR
Whipple (2008)	Traditional lullabies sung by female child accompanied by piano (music therapist)	Pacifier Activated Lullaby (PAL) (in incubator)	65 dB	1 day, 1x daily, 10 minutes	During heel stick procedure
Arnon (2006)*	Lullaby style with Eastern and Western musical elements accompanied by drum and harp (music therapist)	Tape recorded with two speakers 1 meter from infant’s bed (outside incubator)	55–70 dB	3 days, 1x daily, 30 minutes	1 hour after feeding
Calabro (2005)	Lullaby “Brahms Lullaby” and “Sandman” from the CD Music for Dreaming (music therapist)	Cassette player (in incubator)	60–65 dB	4 days, 1x daily, 20 minutes	NR
Standley (2003)	Lullabies sung by female vocalist (music therapist)	Pacifier Activated Lullaby (PAL) (in incubator)	65 dB	1 day, 1x daily, 15–20 minutes	30–60 minutes before afternoon feeding
**Live music**					
Garunkstiene (2014)*	Selection of Lithuanian and traditional Western lullabies (same for live and recorded) (music therapist)	Music therapist 30 cm from infant’s head (outside incubator)	45–50 dB	3 days, 1x daily, 20 minutes	30 minutes after feeding
Loewy (2013)	1.Parent-preferred lullaby, 2. Ocean Disc, 3. Gato Box (music therapist)	1. Live lullaby, 2. Ocean Disc, 3. Gato Box (outside incubator)	55–65 dB	2 weeks, 3x per week, duration of 1 song (approximately 3 minutes)	Morning or afternoon
Schlez (2011)	Simple improvised melodies in lullaby style (music therapist)	Live harp music (outside incubator)	50–65 dB	3 to 5 days1x daily, 30 minutes	30 minutes after afternoon feeding
Arnon (2006)*	Lullaby style wordless blend of Eastern and Western musical elements (music therapist)	Live music with harp and drum, performed 1 or 2 meters from the infant’s bed (outside incubator)	50–70 dB	3 days, 1x daily, 30 minutes	1 hour after feeding

*Garunkstiene (2014) and Arnon (2006) measure both a recorded and live music intervention.

**Table 3:** Results of the effects of music on *physiological parameters*. Results of the effects of music on *physiological parameters* compared between the intervention and control groups. Tabulated results compare the experimental intervention in boldface to the control condition.

Author	Intervention(s) and comparator	(N); Age	Outcome measures	Results
**Recorded Music**				
Wirth (2016)	**1. Recorded lullaby**2. Maternal voice 3. Control	(62) 30–37 weeks GA	HR, RR	A statistically significant difference for HR during and after intervention (p<0.001) and for RR during and after intervention (p<0.001)
Jabraeili (2016)	**1. Recorded lullaby** 2. Maternal voice 3.Control	(66) 29–34 GA	SatO2	A statistically significant difference for SatO2 (p<0.02).
Dorn (2014)	**1.Recorded lullaby** 2.Maternal voice reading 3. Control	(61); 30–37 weeks GA	Cortisol	NS
Garunkstiene (2014)	1.Live lullaby **2.Recorded lullaby** 3.Control	(35); 26–30 weeks GA	HR, SatO2	A statistically significant difference for HR (p<0.001).
Amini (2013)	**1.Lullaby 2.Mozart** 3. Control	(25); 29.4–35 weeks GA	HR, RR, SatO2	Results between groups NR*
Alipour (2012)	**1. Lullaby music with headphone** 2. Silence with headphone 3. Control	(90); 28–36 weeks GA	HR; RR; SatO_2_	NS
Aydin (2012)	**1. Classical music** 2. Control	(26); age NR	HR; RR; SatO_2_	NS
Farhat (2010)	**1. Lullaby** 2. Control	(44); <34 weeks GA	HR; RR; SatO_2_	Statistically significant difference between the groups during the intervention in RR (p = 0.017). NS between the groups after the intervention in RR (p = 0.94). Statistically significant difference between the groups during and after the intervention in SatO2 (p = 0.001 and p = 0.019 resp.)
Keith (2009)	**1.Lullaby** 2.Control	(24); 32–40 weeks GA	HR, RR, SatO_2_	Results between groups NR
Whipple (2008)	**1.PAL** 2.Pacifier 3.Control	(60); 32–37 weeks GA	HR, RR, SatO_2_	NS
Arnon (2006)	1. Live music lullaby 2. **Recorded music lullaby** 3. Control	(31); 25–34 weeks GA	HR; RR; SatO_2_	NS
Calabro (2005)	**1. Lullaby** 2. Control	(22); 34 weeks GA	HR; RR; SatO_2_	NS (HR: p = 0.64 RR p = 0.38 SatO_2_ p = 0.36)
**Live Music**				
Garunkstiene (2014)	**1.Live lullaby** 2.Recorded lullaby 3.Control	(35); 26–30 weeks GA	HR, SatO2	A statistically significant difference for HR (p<0.001).
Loewy (2013)	**1.Lullaby 2.Ocean Disc 3.Gato Box** 4. Control	(272); 32–40 weeks GA	HR; RR; SatO_2_	NS
Schlez (2011)	**1. Harp music therapy with Kangaroo Care** 2. Control: Kangaroo Care	(52) 26–36 weeks GA	HR; RR; SatO_2_	NS
Arnon (2006)	**1. Live music lullaby** 2. Recorded music lullaby 3. Control	(31); 25–34 weeks GA	HR; RR; SatO_2_	Statistically significant difference for HR (p < 0.01).

HR: heart rate, NR: Not reported, NS: Not significant, p-value not reported in the study, PAL: Pacifier Activated Lullaby, RR: respiratory rate, SatO2: Oxygen saturation.

* Results within groups: Statistically significant difference for RR for the lullaby group (p = 0.001) and the Mozart group (p = 0.037).

**Table 4:** Results of the effects of music on *growth and feeding outcomes*. Results of the effects of music on *growth and feeding outcomes* compared between the intervention and control groups. Tabulated results compare the experimental intervention in boldface to the control condition.

Author	Intervention(s) and comparator	(N); Age	Outcome measures	Results
**Recorded music**				
Chorna (2014)	**1.PAL children’s songs sung by mother** 2. Control	(94); 34–36 weeks GA	Feeding rate; length hospitalization; feeding volume and frequency; no of days to full feed; balancing measurements (discharge weight, growth rate, change in salivary cortisol)	Statistically significant increase in feeding rate between the groups after the intervention (p<0.001); oral volume intake (p = 0.001); oral feeds per day (p = 0.001) and faster time to full oral feedings (p = 0.04)
Aydin (2012)	**1. Classical music** 2. Control	(26); NR	Growth (weight, height, head circumference)	NS
Farhat (2010)	**1. Lullaby** 2. Control	(44); <34 weeks GA	Weight gain	NS (p = 0.093)
Lubetzky (2010)	**1. Baby Mozart CD** 2. Control	(20); 30–34 weeks GA	Resting energy expenditure (REE)	Statistically significant reduction in REE between the groups after the intervention (p = 0.03).
Standley (2010)	**1. PAL 1x 2. PAL 3x** 3. Control	(68); 28–32 weeks GA	Days of nipple feeding prior to discharge; discharge weight; weight gain	Results between groups NR
Standley (2003)	**1. PAL** 2. Control	(32); 32 weeks GA	Feeding rate	NS
**Live music intervention**				
Loewy (2013)	**1. Lullaby 2. Ocean Disc 3. Gato Box** 4. Control	(272); 32–40 weeks GA	Caloric intake; Sucks per minute and sucking pattern	Results between groups NR* for caloric intake. Statistically significant difference between the Gato box intervention and control group in sucking behaviour (p = 0.01). Results for Lullaby and Ocean Disc NR.

NR: Not reported, NS: Not significant, p-value not reported in the study, PAL: Pacifier Activated Lullaby.

*Within group results only reported per age group, not per intervention group (32, 34 and 36 weeks GA).

**Table 5:** Results of the effects of music on *behavioural state*, *relaxation outcomes and pain*. Results of the effects of music on behavioural state, relaxation outcomes and pain between the intervention and control groups. Tabulated results compare the experimental intervention in boldface to the control condition.

Author	Intervention(s) and comparator	(N); Age	Outcome measures	Results
**Recorded music**				
Wirth (2016)	**1. Recorded lullaby** 2. Maternal voice 3. Control	(62); 30–37 weeks GA	Activity	Statistically significant less activity for the lullaby group (p < 0.04).
Cardoso (2014)	**1.Recorded lullaby** 2.Recorded lullaby with 25% glucose 3.Control	(80); 31–37 weeks GA	Pain (measured by PIPP)	NS (p = 0.40)
Dorn (2014)	**1.Recorded lullaby** 2.Maternal voice reading 3. Control	(61); 30–37 weeks GA	Rest-activity behaviour	NS
Garunkstiene (2014)	1.Live lullaby **2.Recorded lullaby** 3.Control	(35); 26–30 weeks GA	Behavioural state (from deep sleep to prolonged respiratory pause >8sec)	NS
Alipour (2012)	**1.Lullaby music with headphone** 2. Silence with headphone 3. Control	(90); 28–36 weeks GA	Behavioural state(from quiet sleep to crying)	NS
Aydin (2012)	**1. Classical music** 2. Control	(26); NR	Stress (from no stress to severe stress)	NS
Olischar (2011)	**1.Lullaby** 2. Control	(20); >32 weeks GA	Sleep-Wake-Cycle and Quiet Sleep pattern on aEEG	NS (SWC p = 0.90 QS p = 0.08)
Keith (2009)	**1. Lullaby** 2. Control	(24); 32–40 weeks GA	Frequency of inconsolable crying (>5 minutes crying).	Statistically significant less crying episodes for the lullaby group (p < 0.001).
			Duration of inconsolable crying (in minutes)	Statistically significant difference between the groups for the duration of crying episodes (p < 0.001)
Whipple (2008)	1.PAL 2.Pacifier 3.Control	(60); 32–37 weeks GA	Behavioural state (measured by he Continuous response Digital Interface system Stress	NS
Arnon (2006)	1. Live music lullaby 2. **Recorded music lullaby** 3. Control	(31); 25–34 weeks GA	Behavioural state (from quiet sleep to crying)	NS
Calabro (2005)	1. Lullaby 2. Control	(22); 34 weeks GA	Behavioural State	NS
**Live music**				
Garunkstiene (2014)	**1.Live lullaby** 2.Recorded lullaby 3.Control	(35); 36–30 weeks GA	Behavioural state (from deep sleep to prolonged respiratory pause)	Statistically significant difference in the live lullaby (p = 0.003) for behavioural state.
Loewy (2013)	1.Lullaby 2.Ocean Disc 3.Gato Box 4. Control	(272); >32 weeks GA	Activity level % of quiet-alert time	Results between groups NR*
			Sleeping level % time of active sleep	Increase in positive sleep patterns in Ocean Disc group (p<0.001)
Schlez (2011)	1.Live harp music 2.No music	(52) 26–36 weeks GA	Behavioural State (from deep sleep to prolonged respiratory pause)	NS
Arnon (2006)	**1. Live music lullaby** 2. Recorded music lullaby 3. Control	(31); 25–34 weeks GA	Behavioural State (from quiet sleep to crying)	Statistically significant difference for the live music therapy compared to recorded music therapy and no music therapy after the intervention (p < 0.001).

NR: Not reported, NS: Not significant, p-value not reported in the study, PIPP: Premature Infant Pain Profile

* Within group results: activity level % of quiet-alert time for lullaby group (p<0.05). Increase in positive sleep patterns in Ocean Disc group: p<0.001).

## Results

The search strategy yielded 7744 citations; after duplicates were removed 4893 citations were left for screening. A total of 20 RCTs were included, of which 12 had a parallel group design, 7 a crossover design and 1 a factorial randomized trial design (see Fig 2 PRISMA flowchart).

The total number of subjects was 1128, all born prematurely before 37 weeks, admitted to a NICU and receiving music interventions at ages ranging from 25 to 40 weeks GA. The studies dated from 2003 to 2016 and had been carried out in the USA [4,12,23–26], Israel [27–29], Iran [30–33], Turkey [34], Australia [35,36], Lithuania [37], Germany [38,39] and Brazil [40]. Live music was offered in four studies; recorded music in eighteen studies. Two studies [29,37] offered both live and recorded music interventions.

### Risk of Bias

Thirteen out of 20 studies were rated to have an unclear risk of bias (see Table 1). Eight studies did not report the randomization method. In two studies study personnel was not blinded; twelve other studies did not report on blinding. The outcome assessor was blinded to group allocation in ten studies [4,24–26,28,29,31,35–37]. Four studies published between 2012 and 2015 had a low overall risk of bias [4,26,31,36], which indicates an improvement of the quality of the recent studies.

### Description of the Interventions

#### Recorded music interventions

Eighteen studies [12,23–27,29–40] offered *recorded music interventions* consisting of recordings of lullabies with or without song and classical instrumental music. In nine studies the choice of the music intervention was based on the advice of a certified music therapist; the research team selected the music in the other nine studies. In two studies the music was delivered through headphones [31,40]; four studies used the PAL [12,24–26]; the other twelve studies used speakers in or near the incubator. The decibel levels varied between 40 and 70 dB. The intervention was offered once or 3 times daily for 3 to 60 minutes during a period from 1 to 14 days. The intervention was performed after feeding [27,29–32,37,39]; before feeding [24,26]; in the afternoon [25,34]; between 8 and 9 PM [38]; between 10 AM and 7 PM[33]; during amplitude-integrated EEG recordings [36]; during heel stick procedure [12] and before arterial puncture [40]. Two studies did not report on the timing [23,35] (see Table 2).

#### Live music interventions

Four studies [4,28,29,37] offered *live music interventions* in the form of lullabies selected and sung by a certified music therapist to the accompaniment of a harp, drum, the Ocean Disc, or the Gato Box. The decibel levels varied between 45 and 70 dB. The frequency varied between once daily and thrice weekly for 5 to 30 minutes. The intervention was performed after feeding and one study mentioned it was either in the morning or in the afternoon (see Table 2).

All recorded and live music interventions were in lullaby style and sung by a woman when the lullaby was a song. The music was culturally appropriate, for example studies carried out in Israel made use of both western and eastern musical elements. Five studies [27,30,34–36] used classical music.

## Outcomes

### Physiological Parameters

In fourteen studies the physiological parameters heart rate (HR), respiratory rate (RR), oxygen saturation (SatO2) and cortisol served as outcomes (see Table 3). These were measured before, during and after the intervention by either the investigators or a nurse. Seven of the twelve studies that used a *recorded music intervention* reported no statistically significant difference for any of the physiological parameters between the intervention and control group or condition [12,23,29,31,34,35,38]. Farhat et al. [32] found a significant difference for RR during the intervention (p = 0.017) and for SatO2 during and after the intervention (p = 0.001 and p = 0.019 respectively). The authors did not report the direction of differences. Garunkstiene et al [37] showed a significant decrease in HR after the intervention compared to the control condition (p<0.001). Jabraelili et al [33] reported an statistically significant increase in SatO2 for the lullaby group compared to the control condition (p = 0.02). Wirth et al [39] showed a significant decrease in respectively HR and RR during and after the intervention compared to the control condition (p<0.001 for both HR and RR). Amini et al [30] did not report the between group results.

Four studies measured the effect of a *live music intervention* on physiological parameters. Arnon et al and Garunkstiene et al showed a significant decrease in HR after the intervention (both p<0.001) [29,37]. In the two other studies no significant differences were found [4,28].

### Growth and Feeding Outcomes

Seven studies [4,24–27,32,34] measured growth and feeding outcomes (see Table 4). Six studies measured the effect of a *recorded music intervention*. Lubetzky et al. reported a significant reduction in resting energy expenditure (REE) after the intervention (p = 0.03) [27]. Chorna et al. reported a significant increase in feeding rate (p<0.001), oral volume intake (p = 0.001), oral feeds per day (p = 0.001) and fast time to full oral feedings (p = 0.04) [26]. Three studies did not show a difference and one study [25] did not report intergroup results (see Table 4).

Loewy et al. measured the effect of *live music interventions* on growth and feeding [4]. Intergroup results for caloric intake were not reported. Sucking behaviour in the intervention group receiving the Gato Box intervention had significantly improved relative to the control group (p = 0.01) (see Table 4).

### Behavioural State, Relaxation Outcomes and Pain

Thirteen studies [4,12,23,28,29,31,34–40] measured the effects of music on behavioural state, relaxation outcomes and pain. Outcome measurements included behavioural state, levels of activity and sleep, inconsolable crying, stress and pain (see Table 5). Eleven studies used a *recorded music* intervention; four studies a *live music* intervention (Garunkstiene et al and Arnon et al used both a recorded and live music intervention).

Behavioural state was measured with four different scales. Garunkstiene et al, Schlez et al and Arnon et al used a 7-point behavioural scale [41,42] distinguishing the following states: deep sleep; light sleep; drowsy; quiet awake or alert; actively awake and aroused; highly aroused; upset or crying; prolonged respiratory pause > 8 sec. Alipour et al used a 6-point behavioural scale adapted from Als et al [42] distinguishing six states: quiet sleep, active sleep, drowsy, quiet awake, active awake and crying. Calabro et al assessed the infant’s behavioural state from a Psychological and Behavioural Assessment Form (adapted from Als et al [42]; Hiniker et al [43]; Schoemark et al [9]. Whipple et al [12] measured behavioural state using the Continuous Response Digital Interface (CRDI) system, which distinguishes six behavioural states: deep sleep, light sleep, drowsy, alert, active, and crying. Whipple et al used the CRDI also to measure stress on a nine-point continuum from minimum to maximum stress.

Four studies used *recorded music interventions* but none found a significant effect on behavioural state. Regarding *live music interventions*, Garunkstiene et al (p = 0.003) and Arnon et al (p<0.001) reported a significant improvement in behavioural scores, reflecting a deeper state of sleep.

Wirth et al used the GT3xt accelerometer, a device that tracks body movements, to measure activity during music stimulation. The lullaby group showed significantly less activity during the intervention [39]. Activity-rest behaviour was measured by Dorn et al with an actigraph monitoring device (ActiSleep+, ActiGraph, Pensacola, FL) that monitors activity acceleration on vertical, horizontal and perpendicular axes. No significant difference was found between the *recorded* music and control conditions.

Olischar et al used an amplitude-integrated EEG (aEEG) to monitor the sleep-wake cycles and quiet sleep in infants who received a *recorded lullaby*. Quiet sleep periods did not differ significantly between groups.

Infants in Loewy’s study received a *live* lullaby, the Ocean Disc and Gato Box. The Ocean Disc intervention was associated with a significantly better quality of sleep (p<0.001).

Keith et al. measured the effects of a *recorded intervention* on the frequency and duration of inconsolable crying. For the purpose of that study, inconsolable crying was defined as ‘intense and sustained cry vocalizations, accompanied by high motor activity for 5 minutes or more after interventions to comfort him’. There was a significant decrease in the frequency and duration of crying episodes (both p<0.001).

Stress outcomes were measured by Aydin et al using a non-validated 4 point scale with score categories: no stress symptoms (0 points), mild stress symptoms (1 point), mid-level stress (2 points) and severe stress (3 points). A *live music* intervention was not associated with a significant reduction in stress scores.

Pain was measured by Cardoso et al using the Premature Infant Pain Profile (PIPP), which has been validated to measure acute pain in premature infants. A *recorded music intervention* did not have a significant effect on these pain scores.

## Discussion

This review shows that in three studies offering live music interventions an improvement in sleep quality was reported [4,29,37]. However, sample size varied greatly: Garunkstiene and Arnon used a sample size of respectively 35 and 31 patients, whereas Loewy’s study included 272 patients. The smaller sample sizes could have influenced the positive outcome of the studies. In Loewy’s study, only the patients receiving the Ocean Disc intervention showed an improvement in sleep. In a fourth study offering a live music intervention no behavioural state change was seen, perhaps because the infants simultaneously received kangaroo care, which in itself is an effective intervention. Four studies (Wirth; Garunkstiene both live and recorded; Arnon live) were associated with a significant decline in heart rate. However, seven studies did not show such an association. Better feeding and sucking outcomes were reported in one study using live music and two studies using recorded music [4,26,27]. The latest review on the effect of music interventions in premature infants was published in 2009. We included four [12,24,29,35] out of the nine studies in this review in our updated review; the other five [5,44–47] included too few patients or included term infants. Fifteen out of the twenty RCTs in this updated review were published after 2009, indicating a growing interest in this topic.

### Limitations of the Studies

It was not possible to perform a meta-analysis due to heterogeneity of type and duration of interventions, gestational age of the subjects, and outcome measures. In all included studies, 16 of the 26 outcome measures were addressed only once. Most studies reported their results only expressed as p-values and the authors did not respond to our requests for raw data. The studies included in this review vary in quality. The overall risk of bias was unclear in 13 out of the 20 studies and high in 3 studies. In 11 studies no more than 20 patients per treatment group were included.

Outcomes were measured with a wide range of methods and instruments. For instance, growth and feeding outcomes were measured with 8 different methods, ranging from Resting Energy Expenditure to weight gain and growth. Behavioural state was measured with four different, non-validated instruments. Duration of the intervention varied from 3 to 60 minutes; duration of the study period from 1 day to 3 weeks. In most studies, the intervention was offered after feeding and in all but two studies the intervention was offered once daily (28, 34). Either a music therapist or the research team selected the interventions. It is noteworthy that in the majority of the studies that showed a positive result a certified music therapist had selected the music. This suggests it is advisable for future studies to include the advice of a certified music therapist.

Furthermore, the studies included infants of a wide age range, i.e. although all children were born before 37 weeks GA, some studies provided the children with a music intervention for the duration of their stay in the NICU which resulted in an age range of 25–40 weeks GA. It is not unreasonable to assume that there is a relation between maturity and responses to music, but this was not discussed in these studies. Standley et al. [14] have suggested age guidelines for music interventions, but these recommendations were not adhered to in all studies [28,29,37].

With regard to safety aspects, none of the studies addressed possible adverse effects of the noise levels or the use of headphones (risk of pressure sores) or disinfectants to clean the music equipment, or adherence to NICU hygiene standards. The American Academy of Pediatrics (AAP) has warned that high noise levels may adversely affect newborns’ growth and development. Environmental noise levels should not exceed 40–45 dB and noise levels <35 dB are desired for sleep [48]. Yet, in no more than two studies the noise levels were kept lower than 45 dB [30,37]. Whipple et al [12] did refer to guidelines for sound in the NICU but these were out-dated. Also, overstimulation can be harmful and therefore music interventions should not last 30 minutes or longer without a clear motivation for this duration.

### Limitations of This Review

A limitation to this review could be a possible language bias from the use of studies published in English only. Therefore we performed a post-hoc search and excluded the studies published in English (see S2 File). This search resulted in 113 articles, none of which matched with our inclusion criteria.

### Implications for Practice and Research

First of all, for further studies to be useful, consensus must be reached on the most relevant outcomes, duration and timing of music interventions and how they should be measured. Sleep quality is an important outcome and it might be worthwhile to focus research efforts on measuring sleep-wake cycles through EEGs rather than only observing behavioural state.

Second, Shoemark et al. suggest that a music intervention should be selected on predictable patterns in rhythm, melody and phrasing, gradual changes in tempo in a lullaby, smooth melodic contours and an absence of harmonies [49]. They also suggest a vital role for music therapists in a family-centered approach with positive stimulation from music interventions and interventions including a parent’s voice. Nine studies [12,23–25,32,37–39] in our review used music interventions accompanied by a female voice (Garunkstiene both for the recorded and live intervention) in one study [26] the infant’s mother sung and one study [4] based the music intervention on the preference of both parents. Future studies should take into account the role of the father and place more emphasis on the possible effect of culturally specific music interventions.

In conclusion, taken together, the variation in quality of the studies, age groups, outcome measures and timing of the interventions across the studies makes it impossible to draw strong conclusions on the effects of music on premature infants’ well-being. Still, music interventions might be promising in this respect, especially live music interventions administered by certified music therapists with timing and duration tailored to the infant’s needs. Neonatology staff, music therapists and researchers need to systematically define clinically relevant outcome measures. Furthermore, safety measures for overstimulation should be developed. Future research should focus on performing well-powered RCTs and reporting the between group results.

## Supporting Information

S1 File Review protocol. (DOCX)

S2 File Full list of search terms and databases. (DOCX)

S3 File Cochrane data collection form for intervention reviews: RCTs. (DOC)

S1 Table PRISMA checklist. (DOC)

S2 Table Background characteristics all studies. (DOCX)

## Abbreviations

AAP: American Academy of Pediatrics
dB: decibel
GA: gestational age
HR: heart rate
NICU: neonatal intensive care unit
NIDCAP: Newborn Individualized Development Care and Assessment Program
PAL: Pacifier Activated Lullaby
PIPP: Premature Infant Pain Profile
RCT: randomized controlled trial
REE: resting energy expenditure
RR: respiratory rate
SatO2: oxygen saturation
