# Supplementary material for: Do Hospitalized Premature Infants Benefit from Music Interventions? A Systematic Review of Randomized Controlled Trials
Source: PLoS One. 2016 Sep 8;11(9):e0161848. doi: 10.1371/journal.pone.0161848 (PMC5015899; doi:10.1371/journal.pone.0161848)
Supplement: S1 File — (DOCX) [file pone.0161848.s001.docx]

**Supplement S2**

**Protocol Systematic Review Premature Infants**

**Title**

Do hospitalized premature infants benefit from music interventions? A systematic review of randomized controlled trials

**Authors**

Marianne J.E. van der Heijden, MSc^1^; Sadaf Oliai Araghi, MSc^2^; Johannes Jeekel^3^, MD, PhD; Irwin K.M Reiss, MD, PhD^4^, M.G. Myriam Hunink, MD, PhD ^5,6,7^_,_ Monique van Dijk, PhD^8^

Affiliations: ^1^ Department of Pediatrics, Erasmus MC, Rotterdam, the Netherlands ^2,3^ Department of Neuroscience, Erasmus MC, Rotterdam, the Netherlands ^4^ Department of Neonatology, Erasmus MC, Rotterdam, The Netherlands ^5^ Department of Epidemiology, Erasmus MC, Rotterdam, the Netherlands ^6^ Department of Radiology, Erasmus MC, Rotterdam, the Netherlands ^7^ Department of Health Policy and Management, Harvard T.H. Chan School of Public Health, Boston, MA, USA ^8^ Department of Pediatrics, division of Neonatology and Department of Pediatric Surgery, Intensive care Erasmus MC, Rotterdam, the Netherlands

**Rationale**

Neonatal intensive care units (NICU) around the world increasingly use music interventions. The most recent systematic review of randomized controlled trials (RCT) dates from 2009. Since then, new RCTs have been published. We provide an updated systematic review on the possible benefits of music interventions on premature infants’ well-being.

**Objective**

To conduct a systematic review of randomized controls on the effects of music in preterm infants.

**Methods**

|  | **Inclusion** | **Exclusion** |
| --- | --- | --- |
| **Patients** | Patients born between 24-37 weeks GA and admitted to the NICU* (patients receiving an intervention >37 weeks can be included if they were born <37 weeks) |  |
| **Interventions** | Recorded or live music interventions,  Interventions that required participation of a parent are only accepted if it includes a musical intervention such as singing.  - Music selection: pre-selected by investigator or by therapist;  - Modes of delivery: live music, recorded music, instrumental music, music with song and Pacifier Activated Lullaby (PAL) | Interventions using speech, sounds of the mother’s womb, studies using non-human sounds such as nature sounds.  Interventions using the mother or father’s voice without music.  Interventions comparing different music intervention to other music interventions, for example recorded vs. live music or PAL vs. live music. |
| **Comparison** | Any comparison, as long as the effect of music can be analyzed separately from the control condition. |  |
| **Outcomes** | Unrestricted |  |
| **Study design** | RCTs with a parallel group, cross-over or cluster design | Parallel or group design RCTs with fewer than 10 patients per group.  Cross-over design fewer than 15 per group. |
| **Setting** | NICU* |  |
| **Type of publication** | Scientifically peer-reviewed publications | Unpublished dissertations, conference papers |
| **Year of publication** | Unrestricted |  |
| **Language of publication** | English | Non-English |

NICU: Neonatal Intensive Care Unit

**Search strategy**

- In collaboration with Wichor Bramer, information specialist, Erasmus Medical Center
- **Information sources that will be used:** Electronic databases; specialized journals; trial registers; contact with study authors; check the references in articles
- **Data collection and selection** MvdH and SO will both screen and read the articles. They will use the Cochrane Handbook for Systematic Reviews of Interventions.

**Risk of bias in individual studies**

Cochrane Risk of bias tool

**Data synthesis**

If data is appropriate for quantitative synthesis we will perform a meta-analysis, following the guidelines from the Cochrane Center
