# Supplementary material for: Do Hospitalized Premature Infants Benefit from Music Interventions? A Systematic Review of Randomized Controlled Trials
Source: PLoS One. 2016 Sep 8;11(9):e0161848. doi: 10.1371/journal.pone.0161848 (PMC5015899; doi:10.1371/journal.pone.0161848)
Supplement: S2 File — (DOCX) [file pone.0161848.s002.docx]

**Supplement S3**

**Full list of databases and search terms**

We searched 13 electronic databases and trial registers from their first available date: 1.Cochrane Central Register of Controlled Trials (CENTRAL)

2.MEDLINE (Ovid) (1950 to present)

3.EMBASE (1980 to present)

4.CINAHL (1982 to present)

5.PsycINFO (1967 to present)

6.AMED (The Allied and Complementary Medicine Database) (1985 to present)

7.Web of Science (1945 to present)

8.Scopus (1995 to present)

9.The specialist music therapy research database at [www.musictherapyworld.net](http://www.musictherapyworld.net)

10.CAIRSS for Music

11.ClinicalTrials.gov (<http://www.clinicaltrials.gov/>)

12. Current Controlled Trials (<http://www.controlledtrials.com/> )

13. National Research Register (<http://www.updatesoftware.com/National/>)

Furthermore we hand-searched 12 journals from their first available date:

1. Australian Journal of Music Therapy

2.Canadian Journal of Music Therapy

3.The International Journal of the Arts in Medicine

4.Journal of Music Therapy

5.Journal for Art Therapies in Education, Welfare and Health Care

6.Music Therapy

7.Music Therapy Perspectives

8.Nordic Journal of Music Therapy

9.Music Therapy Today (online journal of music therapy)

10.Voices (online international journal of music therapy)

11.New Zealand Journal of Music Therapy

12.British Journal of Music Therapy

**Search terms**

**Embase**

(music/de OR 'music therapy'/de OR (music OR musical OR musicotherap* OR ((mother* OR maternal) NEAR/3 (sang OR singing OR song OR songs OR lullab*))):ab,ti) AND (prematurity/de OR 'low birth weight'/de OR newborn/exp OR 'newborn care'/exp OR 'newborn monitoring'/de OR incubator/de OR 'neonatal incubator'/de OR 'newborn nursing'/exp OR 'neonatal stress'/de OR 'newborn period'/de OR 'premature labor'/de OR (prematur* OR preterm* OR (pre NEXT/1 (term* OR matur*)) OR (new* NEXT/1 born*) OR newborn* OR neonat* OR infan* OR baby* OR babies OR 'low birth weight' OR 'low birthweight' OR LBW OR VLBW OR ELBW OR ('small for' NEXT/2 (date OR age)) OR SGA OR incubator* OR NICU):ab,ti)

**Medline OvidSP**

(music/ OR "music therapy"/ OR (music OR musical OR musicotherap* OR ((mother* OR maternal) ADJ3 (sang OR singing OR song OR songs OR lullab*))).ab,ti.) AND (exp "Infant, Newborn"/ OR exp "Infant, Low Birth Weight"/ OR "Intensive Care, Neonatal"/ OR "Intensive Care Units, Neonatal"/ OR exp incubators/ OR "Premature Birth"/ OR (prematur* OR preterm* OR (pre ADJ (term* OR matur*)) OR (new* ADJ born*) OR newborn* OR neonat* OR infan* OR baby* OR babies OR "low birth weight" OR "low birthweight" OR LBW OR VLBW OR ELBW OR ("small for" ADJ2 (date OR age)) OR SGA OR incubator* OR NICU).ab,ti.)

**Cochrane**

((music OR musical OR musicotherap* OR ((mother* OR maternal) NEAR/3 (sang OR singing OR song OR songs OR lullab*))):ab,ti) AND ((prematur* OR preterm* OR (pre NEXT/1 (term* OR matur*)) OR (new* NEXT/1 born*) OR newborn* OR neonat* OR infan* OR baby* OR babies OR 'low birth weight' OR 'low birthweight' OR LBW OR VLBW OR ELBW OR ('small for' NEXT/2 (date OR age)) OR SGA OR incubator* OR NICU):ab,ti)

**Web of science**

TS=(((music OR musical OR musicotherap* OR ((mother* OR maternal) NEAR/3 (sang OR singing OR song OR songs OR lullab*)))) AND ((prematur* OR preterm* OR (pre NEAR/1 (term* OR matur*)) OR (new* NEAR/1 born*) OR newborn* OR neonat* OR infan* OR baby* OR babies OR "low birth weight" OR "low birthweight" OR LBW OR VLBW OR ELBW OR ("small for" NEAR/2 (date OR age)) OR SGA OR incubator* OR NICU)))

**Scopus**

TITLE-ABS-KEY(((music OR musical OR musicotherap* OR ((mother* OR maternal) W/3 (sang OR singing OR song OR songs OR lullab*)))) AND ((prematur* OR preterm* OR (pre W/1 (term* OR matur*)) OR (new* W/1 born*) OR newborn* OR neonat* OR infan* OR baby* OR babies OR "low birth weight" OR "low birthweight" OR LBW OR VLBW OR ELBW OR ("small for" W/2 (date OR age)) OR SGA OR incubator* OR NICU)))

**CINAHL**

(MH music OR MH "music therapy" OR AB (music OR musical OR musicotherap* OR ((mother* OR maternal) N3 (sang OR singing OR song OR songs OR lullab*))) OR TI (music OR musical OR musicotherap* OR ((mother* OR maternal) N3 (sang OR singing OR song OR songs OR lullab*)))) AND (MH "Infant, Newborn"+ OR MH "Intensive Care, Neonatal"+ OR MH "Intensive Care Units, Neonatal" OR MH "Infant Warmers" OR MH "Childbirth, Premature" OR AB (prematur* OR preterm* OR (pre N1 (term* OR matur*)) OR (new* N1 born*) OR newborn* OR neonat* OR infan* OR baby* OR babies OR "low birth weight" OR "low birthweight" OR LBW OR VLBW OR ELBW OR ("small for" N2 (date OR age)) OR SGA OR incubator* OR NICU) OR TI (prematur* OR preterm* OR (pre N1 (term* OR matur*)) OR (new* N1 born*) OR newborn* OR neonat* OR infan* OR baby* OR babies OR "low birth weight" OR "low birthweight" OR LBW OR VLBW OR ELBW OR ("small for" N2 (date OR age)) OR SGA OR incubator* OR NICU)) NOT (MH animals+ NOT MH humans+)

**PsycINFO OvidSP**

(music/ OR "music therapy"/ OR (music OR musical OR musicotherap* OR ((mother* OR maternal) ADJ3 (sang OR singing OR song OR songs OR lullab*))).ab,ti.) AND (120.ag. OR exp "Neonatal Development"/ OR "Neonatal Intensive Care"/ OR "Incubators (Apparatus)"/ OR "Premature Birth"/ OR (prematur* OR preterm* OR (pre ADJ (term* OR matur*)) OR (new* ADJ born*) OR newborn* OR neonat* OR infan* OR baby* OR babies OR "low birth weight" OR "low birthweight" OR LBW OR VLBW OR ELBW OR ("small for" ADJ2 (date OR age)) OR SGA OR incubator* OR NICU).ab,ti.)

**PubMed publisher**

(music[mh] OR "music therapy"[mh] OR (music OR musical OR musicotherap*[tiab] OR ((mother*[tiab] OR maternal) AND (sang OR singing OR song OR songs OR lullab*[tiab])))) AND ("Infant, Newborn"[mh] OR "Infant, Low Birth Weight"[mh] OR "Intensive Care, Neonatal"[mh] OR "Intensive Care Units, Neonatal"[mh] OR incubators[mh] OR "Premature Birth"[mh] OR (prematur*[tiab] OR preterm*[tiab] OR pre term*[tiab] OR pre matur*[tiab] OR new born*[tiab] OR newborn*[tiab] OR neonat*[tiab] OR infan*[tiab] OR baby*[tiab] OR babies OR "low birth weight" OR "low birthweight" OR LBW OR VLBW OR ELBW OR small for date*[tiab] OR small for age*[tiab] OR SGA OR incubator*[tiab] OR NICU)) AND (publisher[sb] OR inprocess [sb])

**Google Scholar**

Music|"mother|mothers|maternal singing|song" "premature infant|infants|babies"|"born prematurely"|prematures|preterm|preterms|"pre term|mature|terms|matures|maturely"|"new born"|newborn|newborns|neonate|neonates|neonatal|LBW|VLBW|ELBW|SGA|incubator|NICU

**Search articles in non-English language**

For the extra search on studies that were published in a language other than English we used the following search terms:

'music'/de OR 'music therapy'/de OR music:ab,ti OR musical:ab,ti OR musicotherap*:ab,ti OR ((mother* OR maternal) NEAR/3 (sang OR singing OR song OR songs OR lullab*)):ab,ti AND ('prematurity'/de OR 'low birth weight'/de OR 'newborn'/exp OR 'newborn care'/exp OR 'newborn monitoring'/de OR 'incubator'/de OR 'neonatal incubator'/de OR 'newborn nursing'/exp OR 'neonatal stress'/de OR 'newborn period'/de OR 'premature labor'/de OR prematur*:ab,ti OR preterm*:ab,ti OR (pre NEXT/1 (term* OR matur*)):ab,ti OR (new* NEXT/1 born*):ab,ti OR newborn*:ab,ti OR neonat*:ab,ti OR infan*:ab,ti OR baby*:ab,ti OR babies:ab,ti OR 'low birth weight':ab,ti OR 'low birthweight':ab,ti OR lbw:ab,ti OR vlbw:ab,ti OR elbw:ab,ti OR ('small for' NEXT/2 (date OR age)):ab,ti OR sga:ab,ti OR incubator*:ab,ti OR nicu:ab,ti) NOT [english]/lim

**Forward citation search based on key references**

- Garunkstiene, R.; Baunauskiene, J. et al. (2014) “Controlled trial of live versus recorded lullabies in preterm infants” Nordic Journal of Music Therapy 23 (1): 71-88

- Chorna, O.; Slaughter, J. et al. (2014) “A pacifier-activated music player with mother’s voice improves oral feeding in preterm infants” Pediatrics 133 (3): 462-468

- Loewy, J.; Stewardt, K. et al. (2013) “The effects of music therapy on vital signs, feeding and sleep in premature infants” Pediatrics 131 (5): 902 - 918

- Amini, E.; Rafiei, P. et al. (2013) “Effect of lullaby and classical music on physiologic stability of hospitalized preterm infants: a randomized trial” Journal of Neonatal Perinatology Medicine 6 (4): 295-301

- Olischar, M.; Shoemark, H. et al. (2011) “The influence of music on aEEG activity in neurologically healthy newborns >/=32 weeks’ gestational age” Acta Paediatrica 1—(5): 670-675

- Lubetzky, R.; Mimouni, F. et al. (2010) “Effect of music by Mozart on energy expenditure in growing preterm infants” Pediatrics 125 (1):e24-28

- Farhat, A.; Amiri, R. et al. (2010) “The effect of listening to lullaby music on physiologic response and weight gain of premature infants” Journal Neonatal Perinatal Medicine 3 (2): 103 - 107
