# Supplementary material for: Do Hospitalized Premature Infants Benefit from Music Interventions? A Systematic Review of Randomized Controlled Trials
Source: PLoS One. 2016 Sep 8;11(9):e0161848. doi: 10.1371/journal.pone.0161848 (PMC5015899; doi:10.1371/journal.pone.0161848)
Supplement: S2 Table — (DOCX) [file pone.0161848.s005.docx]

**Supplement S5**

**Background characteristics all studies**

| **Author, year, country** | **Intervention and Comparison** |  |  |  |  | **Outcome measurements** | **Time of measurements** |
| --- | --- | --- | --- | --- | --- | --- | --- |
|  | **Patient population in GA range (mean)** | **N** | **Study design** | **Intervention group** | **Control group or control condition** |  |  |
| Wirth et al. (2016), Germany | 30 – 37 GA | 62 | Parallel RCT with 3 arms | Recorded music: 1.Lullaby  2.Maternal voice | No music, standard care | Physiological parameters (HR, RR); activity | 15 mins before, 30 mins during, 15 mins after intervention |
| Jabraeili et al. (2016), Iran | 29 -34 GA | 66 | Parallel RCT with 3 arms | Recorded music: 1.Lullaby 2. Maternal voice | No music, standard care | Physiological parameters (SatO2) | 10 mins before, 15 min during, 20 mins after intervention |
| Cardoso et al. (2014), Brazil | >31 - <37 GA | 80 | Parallel RCT with 3 arms | Recorded music: 1.Lullaby 2.Lullaby with 25% glucose | 25% glucose, no music | 1.Pain (PIPP) | Before arterial puncture During arterial puncture |
| Chorna et al. (2014), USA | 34- 36 GA | 94 | Parallel RCT with 2 arms | PAL | No music intervention, standard care routine non nutritive sucking and maternal care | Feeding rate | Start and end of intervention |
|  |  |  |  |  |  | Length hospitalization | Day 0 and day 5 |
|  |  |  |  |  |  | Feeding volume and frequency | Start and end of intervention |
|  |  |  |  |  |  | No of days to full feed | Day 0 and day 5 |
|  |  |  |  |  |  | Balancing measurements (discharge weight, growth rate, change in salivary cortisol) | Day 0 and day 5 |
| Dorn et al. (2014), Germany | 30 – 37 GA | 61 | Parallel RCT with 3 arms | Recorded music: 1.Lullaby 2.Maternal voice reading | No acoustic stimulation, standard care | 1. Physiological parameters (cortisol rhythm pattern) | Before intervention: 10 minutes After intervention: 10 minutes On the 1^st^, 7^th^ and 14^th^ day |
|  |  |  |  |  |  | 2.Rest-activity behaviour |  |
| Garunkstiene et al. (2014), Lithuania | 26-30 GA (28.6) | 35 | Cross-over RCT with 3 sequences | 1.Live lullaby 2.Recorded lullaby | No music, standard care | Physiological parameters (HR, SatO2) | Before intervention: 30 minutes During intervention: 20 minutes After intervention: 30 minutes |
|  |  |  |  |  |  | Behavioural State (7-point scale) | Before intervention: 30 minutes During intervention: 20 minutes After intervention: 30 minutes |
| Amini et al. (2013), Iran | 29.4 – 35 GA (32.4) | 25 | Cross-over RCT with 6 sequences | Recorded music: 1.Lullaby 2.Mozart | No music, standard care | Physiological parameters (HR, RR, SatO2) | Before intervention: 10 minutes During intervention: 20 minutes After intervention: 10 minutes |
| Loewy et al. (2013), Israel | ≥ 32 GA (32.9) | 272 | Cross-over RCT with 4 sequences | Live music  1.Lullaby  2.Ocean disc  3.Gato Box | No intervention , standard care | Physiological parameters (HR, RR, SatO2) | Daily: before, during and after intervention |
|  |  |  |  |  |  | Activity level: % of quiet -alert time | Daily: before, during and after intervention |
|  |  |  |  |  |  | Feeding (sucks per minute and sucking pattern) | Daily: during feeding |
|  |  |  |  |  |  | Sleeping (% time of active sleep) | Daily |
|  |  |  |  |  |  | Caloric intake | Daily |
| Alipour et al. (2012), Iran | 28-36 GA (33.6) | 90 | Parallel RCT with 3 arms | Recorded music 1. Lullaby music with headphone 2. Silence with headphone | No music, standard care | Physiological parameters (HR, RR, SatO_2_) | Before intervention: 5^th^ and 10^th^ minute after placing the earphones. During intervention: 5^th^, 10^th^, 15^th^ and 20^th^ minutes After intervention: 5^th^ and 10^th^ minutes |
|  |  |  |  |  |  | Behavioural state (6-point scale) | Before intervention: 5^th^ and 10^th^ minute after placing the earphones. During intervention: 5^th^, 10^th^, 15^th^ and 20^th^ minutes After intervention: 5^th^ and 10^th^ minutes |
| Aydin et al.(2012), Turkey | (NR) | 26 | Parallel RCT with 2 arms | Recorded music 1. Classical music | No music, standard care | Stress symptoms on a 4-point scale | Before intervention: 1 minute. During intervention: 5^th^ minute, 55^th^ minute. After intervention: NR |
|  |  |  |  |  |  | Physiological parameters (HR, RR and SatO_2_) | Before intervention: 1 minute. During intervention: 5^th^ minute, 55^th^ minute. After intervention: NR |
|  |  |  |  |  |  | Growth parameters: weight, height and head circumference | At admission and discharge |
| Olischar et al. (2011), Australia | >32 GA (38) | 20 | Parallel RCT with 2 arms | Recorded music 1. Brahms lullaby | No music, standard care | Sleep-wake-cycle and quiet sleep on a aEEG | aEEG during four sleep-wake-cycles: one before intervention, three after intervention |
| Shlez et al. (2011), Israel | 26-36 GA (32) | 52 | Cross-over RCT with 2 sequences | Live music 1. Harp music therapy with Kangaroo Care | Kangaroo Care without music | Physiological parameters (HR, RR and SatO_2_) | During intervention: every 2 minutes |
|  |  |  |  |  |  | Behavioural states (7-point scale) | During intervention |
| Farhat et al. (2010), Iran | ≤ 34 GA (30.5) | 44 | Parallel RCT with 2 arms 1:1 | Recorded music: 1. Lullaby | No music, standard care | Physiological parameters (HR, RR and SatO_2_) | Before intervention: 10 minutes. During intervention: 20 minutes. After intervention: 10 minutes |
|  |  |  |  |  |  | Weight gain | Daily |
| Lubetzky et al. (2010), Israel | 30-34 GA (NR) | 20 | Cross-over RCT with 2 sequences | Recorded music 1. Baby Mozart CD | No music, standard care | Resting Energy Expenditure (metabolic measurements) | Before intervention: Not measured. During intervention: 3 times during 30 minutes intervention. After intervention: Not measured |
| Standley et al. (2010), USA | 28-32 GA (NR) | 68 | Factorial RCT with 3 sequences | Recorded music  1.PAL 1x  2.PAL 3x | No PAL, standard care | Days prior to nipple feeding | Daily |
|  |  |  |  |  |  | Days of nipple feeding prior to discharge | Daily |
|  |  |  |  |  |  | Discharge weight | Discharge |
|  |  |  |  |  |  | Weight gain | Birth and discharge |
| Keith et al. (2009), USA | 32-40 GA (33) | 24 | Cross-over RCT with 2 sequences | Recorded music 1. Lullaby with nursing intervention: gentle patting, swaddling, providing pacifier and shifting position | No music, standard nursing intervention | Frequency and duration of inconsolable crying | After nursing intervention when the infant continued to cry for 5 minutes |
|  |  |  |  |  |  | Physiological parameters (HR, RR and SatO_2_) and blood pressure | During crying |
| Whipple et al. (2008), USA | 32- 37 GA (NR) | 60 | Parallel RCT with 3 arms | Recorded music 1.PAL | 1. Pacifier only 2. No intervention, standard care | Behavioural states | 3 minutes before intervention. During intervention. 3 minutes after intervention |
|  |  |  |  |  |  | Stress | 3 minutes before intervention. During intervention. 3 minutes after intervention |
|  |  |  |  |  |  | Physiological parameters (HR, RR, SatO_2_) | 3 minutes before intervention. During intervention. 3 minutes after intervention |
| Arnon et al. (2006), Israel | 25-34 GA (29) | 31 | Cross-over RCT with 3 sequences | 1. Live music lullaby 2. Recorded music lullaby | No music, standard care | Behavioural states (7-point scale) | Before intervention: every 5 minutes. During intervention: every 5 minutes. After intervention: every 5 minutes |
|  |  |  |  |  |  | Physiological parameters (HR, RR and SatO_2_) | 30 minutes before, during and after intervention |
| Calabro et al. (2005), Australia | 34 GA (NR) | 22 | Parallel RCT with 2 arms 1:1 | Recorded music 1. Lullaby | No music, standard care | Physiological parameters (HR, RR and SatO_2_) | Before intervention: 10 minutes. During intervention: 20 minutes. After intervention: 15 minutes |
|  |  |  |  |  |  | Behavioural states (22 positive and negative organised states) | Before intervention: 10 minutes. During intervention: 20 minutes. After intervention: 15 minutes |
| Standley et al. (2003), USA | 32 GA (NR) | 32 | Parallel RCT with 2 arms 1: | Recorded music1. PAL | No PAL, standard care | Feeding rate | Before intervention: morning. After intervention: evening |

S4 Legend GA: gestational age, HR: heart rate, NR: not reported, RR: respiratory rate. SatO_2_: saturated oxygen. PAL: pacifier activated lullaby
